# Impact of Progression of Parkinson's Disease on Swallowing Ability and Oral Environment

**DOI:** 10.1155/2021/5571556

**Published:** 2021-04-23

**Authors:** George Umemoto, Shinsuke Fujioka, Yasuyuki Iwasa, Yoshie Ozaki, Kayoko Koga, Kazumi Nishimura, Yoshio Tsuboi

**Affiliations:** ^1^Swallowing Disorders Center, Fukuoka University Hospital, Fukuoka, Japan; ^2^Department of Neurology, Neuro-Muscular Center, NHO Omuta National Hospital, Fukuoka, Japan; ^3^Department of Neurology, Faculty of Medicine, Fukuoka University, Fukuoka, Japan; ^4^Department of Dentistry, Haradoi Hospital, Fukuoka, Japan; ^5^Maruozaki Dental Clinic, Oita, Japan; ^6^Department of Home Care Nursing, School of Nursing, Faculty of Medicine, Fukuoka University, Fukuoka, Japan; ^7^Japanese Red Cross Kyushu International College of Nursing, Fukuoka, Japan

## Abstract

This study investigated the impact of the severity and treatment of Parkinson's disease (PD) on the swallowing ability and oral environment of patients. Swallowing dysfunction increases the aspiration risk and may lead to poor oral health among patients with PD. We investigated the influences of PD progression and drug treatment on the swallowing ability and oral environment using simple noninvasive screening measurements. We recruited 87 patients with PD (mean age, 71.9 ± 8.0 years; mean Hoehn and Yahr score, 2.9 ± 0.9). The PD condition was assessed in each patient using the unified Parkinson's disease rating scale (UPDRS) part III, diet type and oropharyngeal function using the swallowing disturbances questionnaire (SDQ), maximum bite force (MBF), tongue pressure (TP), and oral bacterial count (OBC). Levodopa equivalent daily dose (LEDD) was also calculated for 56 participants. Based on an SDQ score of ≥11, 29.5% of patients were dysphagic, but almost all were still on a regular diet. The SDQ score was positively correlated with disease duration (*rho* = 0.228, *p*=0.047) and UPDRS part III score (*rho* = 0.307, *p*=0.007) but was negatively correlated with OBC (*rho* = −0.289, *p*=0.012). OBC was significantly higher among patients with an SDQ score of <11 (nondysphagic) (*p*=0.01), and the SDQ score was lower in patients with higher OBC requiring professional oral care (*p*=0.03). However, OBC was also negatively correlated with LEDD (*rho* = −0.411, *p*=0.004). These results indicated low self-awareness of dysphagia among the participants and an association between dysphagia and PD progression. Moreover, the oral environment could have deteriorated with swallowing dysfunction. Patients and clinicians should be aware that higher LEDD can increase xerostomia and associated deficits in oral health.

## 1. Introduction

Swallowing function deteriorates with the progression of Parkinson's disease (PD), necessitating careful adjustment of diet and monitoring of oral health. Swallowing dysfunction (dysphagia) among patients with PD is often evaluated using video fluoroscopic swallowing study (VFSS) or fiberoptic endoscopic evaluation of swallowing (FEES), and VFSS in particular provides detailed information on the oral and pharyngeal stages of swallowing dysfunction [1, 2]. However, these tests are costly and often not available to primary care clinicians. Therefore, more convenient dysphagia screening methods are needed. The swallowing disturbance questionnaire (SDQ) is a 15-item self-reported measure of specific dysphagia symptoms and their frequency [3]. Moreover, patients with PD have many oral health problems in addition to dysphagia, such as reduced number of teeth, more dental caries, poor periodontal health, chewing difficulties, and denture discomfort due to impaired dexterity of arms and fingers for self-care [4]. However, few studies have investigated the influence of physical dysfunction in patients with PD on diet or oral care ability. This study investigated the impact of PD severity and treatment on the swallowing function and oral environment by examining the associations among SDQ scores, unified Parkinson's disease rating scale (UPDRS) part III score, multiple measures of oral environment and function, and levodopa equivalent daily dose (LEDD).

## 2. Methods

### 2.1. Patients Selection

We recruited 87 patients with PD (mean age, 71.9 ± 8.0 years; mean Hoehn and Yahr score, 2.9 ± 0.9). All patients had been diagnosed with PD and received treatment from medical institutions, excluding eight patients who had missing data in the examination. Thirty-one patients with PD (22 men and 9 women; mean age, 71.0 ± 8.0 years; mean Hoehn and Yahr score, 2.6 ± 0.9) who attended the meeting of the Japan PD Association of Fukuoka City on 27 May 2017 were evaluated using UPDRS part III. They completed the SDQ and underwent measurements of occlusal condition, bite force (BF), tongue pressure (TP), and oral bacterial count (OBC) (Table 1). Next, 56 patients with PD (27 men and 29 women; mean age, 72.4 ± 7.2 years; mean Hoehn and Yahr score, 3.1 ± 0.8) who attended the meetings of the Japan PD associations of Kitakyushu City on 23 February 2019 and Iizuka City on 23 March 2019 were examined with the addition of medical regimens for calculation of LEDD. This study was approved by the ethics committee of Fukuoka University Hospital (approval number: 2018M049) and written informed consent was obtained from all participants on the survey days.

### 2.2. Clinical Parameters

UPDRS part III, SDQ, BF, TP, and OBC results were collected from a total of 87 participants and LEDD from 56 participants. Physical examinations were performed for all participants, and body weight, height, and body mass index (BMI) were recorded. The regular diet type was classified into three of seven levels based on the functional oral intake scale (FOIS) [5]. The participants belonged to the following three of the seven categories: Level 0, tube dependent; Level 6, total oral intake with no special preparation but must avoid specific foods or liquid items; Level 7, total oral intake with no restrictions. Neurologists performed assessments using the UPDRS part III score and LEDD; dentists measured BF, TP, and OBC; and nurses helped complete physical examinations, SDQ, and FOIS. The dentists were well-trained for dysphagia rehabilitation and conducted the dental evaluation using EI, BF, OBC, and the evaluation of dysphagia using TP.

Medical history including disease duration was recorded and UPDRS part III was administered by two neurologists, generally in the “on state” after each dose of medication, to assess disease severity. Usual medications for PD were checked for the next 56 participants, and LEDD [6] was calculated to investigate its influence on oral conditions.

The SDQ is a self-reported questionnaire comprising 15 items. Fourteen items are rated from 0 (no disability) to 3 (severe disability), and the final item is a “yes/no” question in which “yes” is scored 2.5 points and “no” is scored 0.5 points. The reliability and validity of the SDQ Japanese version (SDQ-J) have been confirmed [7]. The participants were divided into two subgroups, a nondysphagic subgroup scoring <11 and a dysphagic subgroup scoring ≥11.

The Eichner index (EI) was used as an evaluation of occlusal condition based on existing natural tooth contacts between the maxilla and mandible in the bilateral premolar and molar regions. BF was recorded using the Bite Force Analyzing System (GC, Tokyo, Japan) [8]. This method uses a sheet that undergoes a color-developing chemical reaction when bitten. From the bite pattern, a contact area and balance of an occlusal load are measured with the aid of a computerized analysis system. Each participant was instructed to bite down on a Dental Prescale II sheet (GC) as hard as possible for 3 s, and the test was conducted in triplicate. The average result from the three tests was recorded for analysis. Reduced BF is defined as <200 N [9].

Maximum TP (MTP) was measured using a probe with a small air balloon pressurized to 19.6 kPa (JM-TPM; JMS, Hiroshima, Japan) [10]. The participants were required to compress the balloon onto the palate with their tongue for approximately 7 s while applying maximum effort. The resulting increase in the inner pressure of the balloon was measured and recorded as MTP. This test was also repeated three times, and the mean value was recorded for analysis. Decreased TP was defined as <30 kPa [9].

The number of oral bacteria, measured as OBC, was determined using a bacteria detection apparatus (Panasonic Healthcare, Tokyo, Japan) 2 h after lunch and regular oral care [11]. A sterilized swab was pressed on the dorsal surface of the tongue with a constant force of 20 g using a device with the bacteria detection apparatus. The swab was rubbed back and forth three times over a 1 cm distance. The number of bacteria was quantified (in cfu/mL) using the dielectrophoretic impedance measurement technique. The number of oral bacteria was classified into seven levels as follows: Level 1, <10^5^; Level 2, 10^5^–10^6^; Level 3, 10^6^–10^6.5^; Level 4, 10^6.5^–10^7^; Level 5, 10^7^–10^7.5^; Level 6, 10^7.5^–10^8^; and Level 7, >10^8^. The participants were divided into two groups: those > Level 5, indicative of the need for professional oral care, and those ≤ Level 5 [9].

### 2.3. Statistical Analysis

The relationships among BF, TP, OBC, and BMI were evaluated using Pearson's correlation, and other relationships were evaluated using Spearman's rank-order correlation. Mean values of subgroups with SDQ scores of <11 or ≥11 and subgroups with OBC of >Level 5 or ≤ Level 5 were compared using independent samples *t*-tests. All statistical analyses were performed using SPSS version 13.0 J for Windows (SPSS, Inc., Chicago, IL, USA). *p* < 0.05 (two-tailed) was considered statistically significant for all tests.

## 3. Results

The mean UPDRS part III score, BMI, FOIS, SDQ, BF, TP, and OBC from a total of 87 participants are summarized in Table 1. In the first 31 participants, there was a significant positive correlation between disease severity, as measured using UPDRS part III score, and swallowing dysfunction, as measured using SDQ (*rho* = 0.497, *p*=0.016). However, the SDQ score was negatively correlated with OBC (*rho* = −0.479, *p*=0.021). Both BF and TP were lower in the patients than in healthy volunteers (543.0 ± 298.7 N and 31.9 ± 8.9 kPa, resp.) in previous studies [12, 13]. There were significant positive correlations between TP and BF (*r* = 0.540, *p*=0.002) and between BF and EI (*rho* = 0.407, *p*=0.021) but not between TP and EI (*rho* = 0.295, *p*=0.09).

**Figure 1:**
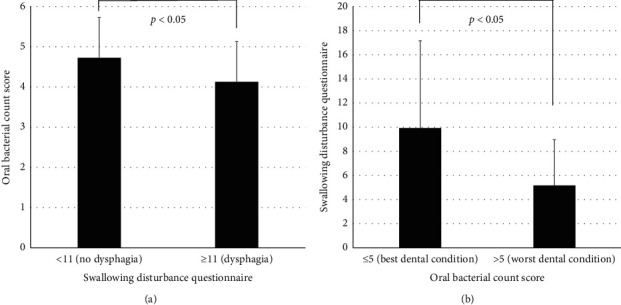
(a) Difference in oral bacteria count (OBC) between patients with an SDQ score of <11 and ≥ 11 among the next 56 participants. (b) Difference in SDQ scores between patients with OBC exceeding Level 5 or ≤ Level 5 among the next 56 participants.

Among the next 56 participants, 13.8% of participants were at Hoehn and Yahr stages 4/5, and 16.9% had a BMI of <20 kg/m^2^, both of which are clinical predictors of dysphagia [14]. However, there were no significant differences in any of the measured values between participants meeting or not meeting these predictive thresholds (Table 1). Most participants were following FOIS Level 6-7 dietary regimens, indicating no special preparation, diet, or avoidance of specific foods or liquid items, and only one participant required tube feeding. However, 29.5% of the participants had an SDQ score of ≥11, indicating likely dysphagia [3]. OBC was significantly higher in the subgroup with an SDQ score of <11 than in the subgroup with an SDQ score of ≥11 (*p*=0.01; Figure 1(a)), whereas the SDQ score was significantly lower among 14.3% of participants who exceeded OBC Level 5 than in those with an OBC of ≤Level 5 (*p*=0.03; Figure 1(b)).

As in the first 31 participants, there were significant positive correlations between swallowing dysfunction, as assessed using SDQ and UPDRS part III (*rho* = 0.307, *p*=0.007; Table 2) as well as between SDQ score and disease duration (*rho* = 0.228, *p*=0.047). Notably, OBC was not correlated with disease duration or UPDRS part III score but was negatively correlated with SDQ score (*rho* = −0.289, *p*=0.012) and LEDD (*rho* = −0.411, *p*=0.004), suggesting an effect of medication on OBC and the oral environment.

## 4. Discussion

While FEES and VFSS are reliable methods for assessing swallowing dysfunction in patients with PD, the SDQ has also been used effectively [15, 16]. For instance, Yamamoto et al. reported a sensitivity of 77.8% and specificity of 84.6% for identifying dysphagia using SDQ-J [7] and diagnosed 24.6% of patients with dysphagia according to a score of ≥11. This result of 24.6% is close to the result of 29.5% of patients with an SDQ score ≥11 in the present study. However, most of these patients were still receiving a normal or only slightly adjusted diet type (Levels 6-7 on FOIS), which indicated a substantial gap between subjective dysphagia complaints and preventive measures against aspiration, as well as generally low self-awareness of dysphagia. Furthermore, the frequency of subjective dysphagia complaints is expected to be lower than that detected objectively as Kalf et al. reported that patients with PD without subjective symptoms had a high frequency of objective swallowing abnormalities and that many of these patients would not report swallowing difficulties unless asked [17]. There, the prevalence of dysphagia may be even greater than the 29.5% defined using SDQ.

We found a significant positive correlation between the UPDRS part III score and SDQ score, indicating that disease progression is associated with more severe swallowing difficulties, corroborating the results of previous studies [18, 19]. Unexpectedly, however, we found a significant negative correlation between SDQ score and OBC in the 87 participants, suggesting that dysphagia progression may reduce the number of oral bacteria in patients with PD despite their limited ability to maintain oral hygiene by themselves [20]. In contrast, Ikeda et al. reported that OBC was significantly reduced by oral care from Level 5 to Level 4 using the same apparatus as that used in this study and suggested that the degree of xerostomia as a result of polypharmacy may affect oral care results [21]. Based on the results of the first 31 participants, we suspected a reduction of oral bacteria caused by xerostomia at Level 4, with no need of professional oral care, and therefore checked usual medications for PD for the next 56 participants. Indeed, OBC was also negatively correlated with LEDD. It is thus possible that xerostomia from medication prevented the efficient collection of oral bacteria from the dorsal surface of the tongue.

The significant negative correlation between OBC and LEDD supported our hypothesis that increasing doses of dopaminergic medication induces xerostomia and the consequent reduction in the number of oral bacteria collected from the tongue. Consistent with this suggestion, a previous study reported that patients with PD produced significantly less saliva and that decreased production of saliva correlated with the dose of levodopa [22]. Barbe et al. also reported an association between subjective dysphagia and xerostomia and that LEDD increased the occurrence of xerostomia [23]. However, the direct effect of dopamine on the sympathetic innervations of the salivary glands that cause a decrease in salivation is unknown. Levodopa was found to be an important contributor to the decreased salivation. The increase in the synaptic levels of dopamine acting on the dopamine D2 receptors on postganglionic neurons of the submandibular ganglion may activate *K*+ channels to hyperpolarize and disrupt neurotransmission.

Dopaminergic medication does not necessarily improve swallowing function. While Müller et al. reported that higher LEDD was associated with improved dysphagia among patients with PD [24], other studies have suggested that dysphagia in PD is largely resistant to dopaminergic stimulation [25, 26]. In the present study, LEDD showed no significant correlation with any swallowing-related variables except OBC.

Decreased TP, defined as <30 kPa [9], was noted in 44.8% of all participants, and reduced BF, defined as <200 N [9], was noted in 51.7% of all participants. There was a significant correlation between TP and BF and between BF and EI in the first 31 participants. In the next 56 participants, there was a significant correlation between TP and age but not between BF and age. Utanohara et al. reported the mean values of TP from selected age groups but reported no dysphagic symptoms [13]. However, BF may not show a direct correlation with age due to the influence of other factors such as the number of existing teeth and the presence of dentures. These findings suggest the importance of interprofessional collaboration for treating PD.

This study has several limitations. First, swallowing dysfunction was evaluated using a self-reported questionnaire and several simple physical measurements rather than more invasive methods. However, it was difficult to request more invasive examinations as the patients were volunteers from a self-help patient group. Although VFSS and (or) FEES could provide more definitive conclusions, the measures used are relatively simple to acquire and provide valuable information for clinical dentistry. Second, we have no definitive proof that LEDD leads to xerostomia as suggested by the decreased OBC. Moreover, many participants took various medications in addition to antiparkinsonian drugs such as antihypertensive, anticoagulation, gastric coating, constipation, and anxiolytic drugs which can cause xerostomia. The information of prescribed drugs was collected based on self-enumeration, and it was difficult to confirm the accurate dosage. If we subclassify the prescribed drugs, it will be hard to perform statistical analysis due to the reduced number. Thus, quantitative measurements of salivation should be conducted to confirm or refute this notion.

Although 29.5% of the participants were defined as dysphagic, most were still on normal or only slightly adjusted diets. These results indicate low self-awareness of dysphagia and underscore the need for more vigilant monitoring of diet type based on objective assessments to reduce the incidence of aspiration. Moreover, the reduction of OBC with greater SDQ score and increasing LEDD suggest that both the patients with PD and neurologists should be informed of possible xerostomia induced by dopaminergic medication. Screening for xerostomia and oral care and moisture are recommended for patients with PD suspected to have dysphagia based on SDQ scores of ≥11 and treated with high doses of levodopa.

## 5. Conclusions

These results indicate low self-awareness of dysphagia among patients with PD and highlight the importance of careful diet-type monitoring and adjustment to prevent aspiration. Moreover, patients and clinicians should be alert for deterioration in oral health with swallowing dysfunction and be aware that higher dopaminergic medication dose (LEDD) may impair the oral environment by inducing xerostomia. Screening for xerostomia and oral care and moisture are recommended for patients with PD with dysphagia who receive high doses of levodopa.

## Figures and Tables

**Table 1:** Characteristics of the first 31 and next 56 participants, and mean values and scores.

	The first 31 participants	The next 56 participants
Age (years)	71.0 ± 7.5	71.9 ± 8.0
Male:Female	22 : 9	49 : 38
Disease duration (years)	8.9 ± 6.1	9.7 ± 6.5
Hoehn and Yahr stage	2.6 ± 0.9	2.9 ± 0.9
UPDRS part III	31.7 ± 19.9	40.4 ± 17.7
BMI (kg/m^2^)	22.5 ± 2.6	22.5 ± 2.8
FOIS	7.0 ± 0.2	6.9 ± 0.7
SDQ	10.7 ± 7.8	9.2 ± 7.0
Bite force (N)	307.9 ± 237.0	302.0 ± 358.7
<200 N	12/31 (38.7%)	33/56 (58.9%)
Tongue pressure (kPa)	26.8 ± 12.0	28.7 ± 9.5
<30 kPa	15/31 (48.4%)	24/56 (42.9%)
Oral bacteria count score	4.2 ± 1.3	4.5 ± 1.1
>5	5/31(16.1%)	7/56 (12.5%)
Eichner index (number)	A1,3; A2,5, A3,3	
	B1,6; B2,9; B3,3, B4,1	
	C1,1	
LEDD (mg)		707.2 ± 428.9

UPDRS, Unified Parkinson's Disease Rating Scale; BMI, body mass index; FOIS, Functional Oral Intake Scale; SDQ, Swallowing Disturbances Questionnaire; LEDD, levodopa equivalent daily dose.

**Table 2:** Correlations between each measured value and score in the next 56 participants.

	Age (years)	Disease duration (years)	Hoehn and Yahr stage	UPDRS part III	BMI (kg/m^2^)	SDQ	LEDD (mg)
SDQ	*rho* = −0.067, *p*=0.554	*rho* = 0.228, *p*=0.047^*∗*^	*rho* = 0.183, *p*=0.153	*rho* = 0.307, *p*=0.007^*∗∗*^	*rho* = 0.077, *p*=0.538		*rho* = 0.126, *p*=0.372
Bite force (N)	*rho* = −0.113, *p*=0.323	*rho* = −0.163 *p*=0.155	*rho* = 0.099, *p*=0.441	*rho* = −0.010, *p*=0.934	*r* = 0.124, *p*=0.326	*rho* = −0.028, *p*=0.809	*rho* = -0.044, *p*=0.759
Tongue pressure (kPa)	*rho* = −0.238, *p*=0.037^*∗*^	*rho* = −0.021 *p*=0.852	*rho* = −0.090, *p*=0.481	*rho* = −0.187, *p*=0.100	*r* = 0.138, *p*=0.273	*rho* = −0.139, *p*=0.223	*rho* = 0.131, *p*=0.360
Oral bacterial count score	*rho* = 0.014, *p*=0.902	*rho* = −0.132 *p*=0.252	*rho* = 0.061, *p*=0.636	*rho* = −0.080, *p*=0.483	*r* = −0.007, *p*=0.936	*rho* = −0.289, *p*=0.012^*∗*^	*rho* = −0.411, *p*=0.004^*∗∗*^

^*∗*^ *p*=<0.05, ^∗∗^*p* < 0.01. UPDRS, Unified Parkinson's Disease Rating Scale; BMI, body mass index; FOIS, Functional Oral Intake Scale; SDQ, Swallowing Disturbances Questionnaire; LEDD, levodopa equivalent daily dose.

## Data Availability

The data used in the study are available from the corresponding author on request.
